# Rush Medical College

**Published:** 1859-07

**Authors:** 


### EDITORIAL.

### RUSH MEDICAL COLLEGE.

The arrangements for filling the vacancies in the faculty of this institution are complete. The corps of professors will be made up as follows:

Prof. of Surgery, Daniel Brainard, M.D.

Prof. of Chemistry and Pharmacy, James V. Z. Blaney, M.D.

Prof. of Surgical Anatomy, Joseph W. Freer, M.D.

Prof. of Obstetrics and Diseases of Women and Children, DeLaskie Miller, M.D.

Prof. of Theory and Practice of Medicine, J. Adams Allen, M.D.

Prof. of Physiology and Pathology, A. S. Hudson, M.D.

Prof. of Materia Medica, Ephraim Ingalls, M.D.

Prof. of Descriptive Anatomy, Robert Rea, M.D.

Of the gentlemen whose names have not before appeared in connection with the college, it is only necessary to say that all are eminently qualified. Several of these are already well known as medical teachers:—Prof. Allen for his connection with the University of Michigan. Prof. Hudson for his connection with the Iowa University, of Iowa. Dr. Rea was formerly Demonstrator of Anatomy in the Ohio Medical College, and has given several courses of lectures on anatomy and physiology. Dr. Miller is a leading practitioner in his department in the city; President of the Chicago Academy of Medical Sciences; and already favorably known to the readers of the *Journal*. Dr. Ingalls is a graduate of Rush Medical College, who has, with great diligence and success, cultivated the branch which he is to teach.

The College Clinic, the Marine Hospital, and the City Hospital, will all afford their advantages for clinical instruction. It is the determination of the faculty to spare no effort nor expense to place Rush Medical College on a par, so far as regards the means of instruction, and the size and character of its classes, with the first institutions of this country.

